# Chinese handwriting performance in preterm children in grade 2

**DOI:** 10.1371/journal.pone.0199355

**Published:** 2018-06-19

**Authors:** Hui-Ning Shih, Wen-Hui Tsai, Shao-Hsia Chang, Chung-Ying Lin, Rong-Bin Hong, Yea-Shwu Hwang

**Affiliations:** 1 Department of Occupational Therapy, College of Medicine, National Cheng Kung University, Tainan, Taiwan; 2 Department of Pediatrics, Chi Mei Medical Center, Tainan, Taiwan; 3 Graduate Institute of Medical Sciences, College of Health Sciences, Chang Jung Christian University, Tainan, Taiwan; 4 Department of Occupational Therapy, I-Shou University, Kaohsiung, Taiwan; 5 Department of Rehabilitation Sciences, Faculty of Health and Social Sciences, The Hong Kong Polytechnic University, Hung Hom, Hong Kong; 6 Department of Physical Medicine and Rehabilitation, Chi Mei Medical Center, Tainan, Taiwan; 7 Department of Physical Medicine and Rehabilitation, National Cheng Kung University Hospital, College of Medicine, National Cheng Kung University, Tainan, Taiwan; Waseda University, JAPAN

## Abstract

**Background:**

First graders born prematurely perform poorly on handwriting speed and legibility. However, whether there are specific legibility factors in which preterm children demonstrate difficulty remains unknown. In addition, handwriting performance beyond the first grade and the influence of sex on handwriting performance in preterm children are still unclear. We aimed to investigate the influence of prematurity and sex on multiple dimensions of handwriting in grade two and to identify the contributors to performance.

**Methods:**

Sixty-three preterm (34 boys and 29 girls) and 67 full-term (27 boys and 40 girls) peers in grade two were included. Class teachers were asked to complete the Chinese Handwriting Evaluation Form. A subgroup of 39 preterm children received assessments on intelligence, visual perception, tactile and kinesthetic sensation, and fine motor skills. Their inattention behavior was rated using a maternal self-report with a behavioral scale.

**Results:**

Boys born prematurely exhibited poorer performance in the speed dimension than full-term boys (p = 0.008), whereas there was comparable performance in the two groups of girls (p = 0.221). In the dimensions related to legibility, preterm boys (32.4%) had a higher percentage of children with difficulty in the construction dimension than the other groups (preterm girls: 6.9%, full-term boys: 7.4%, full-term girls: 5.0%). However, no group difference was found in the dimensions of accuracy and directionality. Of the sensory-perceptual-motor factors, attention was the most significant predictor of accuracy in performance (p = 0.046) and speed dimensions (p = 0.001) in preterm children.

**Conclusions:**

Boys appear to be vulnerable to the adverse impacts of preterm birth in terms of performance in the dimensions of speed and construction in grade two. Based on the significant contribution of attention to handwriting performance in preterm children, assessment and intervention in the area of attention is strongly suggested for preterm children with handwriting problems.

## Introduction

According to the World Health Organization [1], preterm children are defined as those born at less than 37 weeks of gestation. Due to preterm birth, they generally have a difficult time after birth, including prolonged hospital stays and complicated medical conditions. Multiple factors such as prematurity, complications, and medically disadvantageous experiences in early life lead to a delay or deficit in neurodevelopmental outcomes in this population. Even in preterm children without significant neurological impairments [2–5] or those born at an older gestational age, such as moderate-late preterm (i.e. 32^+0^–36^+6^ weeks of gestation) [6, 7], studies also have found that these preterm children exhibit poorer performance in developmental outcomes compared to their full-term peers. Such deficits continuously and negatively impact their academic skills and performance [8–10], and this likely includes handwriting [11, 12].

Some evidence has shown that first graders born prematurely have worse handwriting performance than their full-term peers. In one study, researchers found that compared to 17.1% of full-term peers, 35.5% of preterm children (≤ 33 weeks gestation) with the absence of neurodevelopmental impairment (i.e., moderate-severe cerebral palsy, cognitive delay) were rated as below average or as having delayed handwriting by class teachers [12]. Another study further found that more preterm children in the first grade obtain inferior ratings (needs improvement/very poor) by teachers in both writing legibility (39.5% vs. 22.0%) and speed (44.7% vs. 23.7%) as compared to their classmates born at term [11]. Similarly, through a direct assessment using the Evaluation Tool of Children’s Handwriting-Manuscript (ETCH-M), preterm children have been shown to demonstrate poorer legibility and to write letters and words slower than their full-term classmates [11]. However, the handwriting performance of first graders seems to be unstable [13–15]. For example, one longitudinal and cross-sectional study [15] indicated that the handwriting quality of children improved from the first to second semester in grade two but remained stable in the two semesters of grade three. That is, children may reach their final quality level after the second semester of grade two. Therefore, further investigation on the handwriting performance of preterm children beyond the first grade is needed.

A number of studies have shown that the impact of preterm birth on brain structures and developmental outcomes is sex-different. Compared to preterm-born girls, boys born prematurely have lower scores in developmental assessments, a higher risk of developmental delay [4, 16], and poorer executive outcomes at school age [17]. Preterm boys also demonstrate more adverse brain development (e.g., reduced white matter volume, larger hippocampus density) than preterm girls [18], even up to school age [16, 19]. So far, only one study by Feder and colleagues (2005) investigated the influence of sex on handwriting performance in preterm children. They briefly reported that girls had better legibility in the letter and number handwriting tests than boys in both the preterm and full-term groups, and there was a similar trend in both groups. However, they did not provide the data or separately compare the handwriting performance of boys and girls born prematurely with their same-sexed peers born at term [11]. Therefore, whether male vulnerability to preterm birth also exists in handwriting performance is still unclear.

Handwriting performance is often assessed in terms of two dimensions in the literature: legibility and speed. Legibility consists of a variety of elements such as errors in letter formation, reversals of letters, spacing between letters and words, letter size, slant, and alignment [20, 21]. In fact, through factor analyses, studies indeed have found that these elements of legibility can be separately grouped into a few factors. In the alphabetic system, four factors (letter formation, spacing, alignment, size) have been differentiated [22]. Similarly, in the Chinese handwriting of school-aged children, three factors have been identified, including construction (e.g., spacing between characters, size of characters), accuracy (e.g., adding or missing strokes), and directionality (e.g., reversal of components) [23, 24]. These results infer that children may have illegible handwriting due to difficulties in specific legibility factors. To our knowledge, no research has investigated the handwriting quality of preterm children in terms of these factors.

Handwriting is a complex skill. The maturation and integration of somatosensory (tactile, proprioceptive, kinesthetic), visual perceptual, fine motor, visual-motor integration (VMI), and cognitive functions (e.g., sustained attention, working memory) are considered to be requisites for proficient handwriting [25, 26]. However, the contribution of each component to handwriting performance is not conclusive. The relationship between each performance component and handwriting performance seems to vary with a child’s age [27], handwriting abilities [20, 28], and diagnosis [29, 30]. In the preterm population, only one study by Feder et al. (2005) has explored the contribution of a variety of sensorimotor components to handwriting performance in preterm children [11]. Their results revealed that visual perception was the best predictor for handwriting legibility, whereas fine motor coordination, VMI, and tactile and proprioceptive discrimination were not. [11]. However, the relationship between visual perception and legibility factors remains unclear.

Recently a standardized, teacher-reported handwriting questionnaire, the Chinese Handwriting Evaluation Form (CHEF), was developed by Taiwanese researchers [31]. Its psychometric properties have been validated in first and second graders. The CHEF consists of multiple dimensions of handwriting, including accuracy, construction, directionality (three factors of legibility), speed, and pencil grasp. Therefore, in the present study, we aimed to compare the performance of preterm- and full-term children in multiple dimensions of handwriting using the CHEF. There were three hypotheses in this study: Firstly, the second graders born prematurely were expected to perform worse than their full-term peers in one or more dimensions of the CHEF. Secondly, the impact of prematurity on handwriting was posited to be sex-different. Furthermore, the ability in one or more handwriting components (sensory, visual perceptual, attention, and fine motor functions) was predicted to significantly contribute to handwriting performance in preterm children.

## Methods

### Participants

This study was conducted from November 2014 to June 2015. Two groups of preterm and full-term children were included. The inclusive criteria for the preterm group included (1) born at < 37 weeks of gestation, (2) aged between 7–8 years old, (3) studying in grade 2, and (4) having native Chinese speaking parents. Preterm children who had (1) congenital anomalies, (2) genetic or chromosome abnormalities, (3) auditory and visual problems that cannot be corrected, (4) a diagnosis of neurodevelopmental disorders such as cerebral palsy, attention deficit hyperactivity disorder, autism spectrum disorder, and intellectual disabilities, or (5) a history of significant injuries in the neuromuscular system of the trunk or upper extremities were excluded. Two methods were used to recruit preterm children. One was to identify potential preterm children from the preterm baby registration records of a medical center in Tainan City, Taiwan. A research assistant invited parents to participate in the study by telephone. The other method involved recruiting preterm children through a research advertisement given to the parents of second-grade children attending elementary schools in Tainan City.

The full-term children eligible for participation were (1) born at 37–42 weeks of gestation and were above 2500 grams in weight, (2) typically developing, defined as without any diagnoses or intervention related to developmental delay after birth, and (3) having native Chinese speaking parents. The exclusion criteria for full-term children were the same as those of the preterm children. The elementary schools in each geographic area of Tainan City were randomly selected for contact using a table of random numbers. Of the contacted schools, 24 second-grade class teachers from 7 elementary schools agreed to participate. Parents of 5 randomly selected children in each class were invited to participate.

The study was approved by the Institutional Review Board of Chi Mei Medical Center. Written informed consent was obtained from all parents before data collection. We also asked for the written informed consent of the children who came to our department for evaluation.

### Measures

#### Chinese Handwriting Evaluation Form (CHEF)

The CHEF has two versions: preschool and school. The school version consisting of 25 item questions to evaluate the handwriting performance of first and second graders was used in this study [31]. The class teachers were asked to rate the child’s handwriting performance on each question using a 5-point Likert scale (1: never matching to 5: always matching). The item questions are classified into 5 dimensions of handwriting performance, including construction (8 questions), accuracy (5 questions), speed (4 questions), pencil grasp (6 questions), and directionality (2 questions), determined by the results of the factor analyses. The construction dimension includes the items related to the size, spacing, and alignment of characters and components. The accuracy dimension examines the malformation of characters (i.e., incorrect figuration of components, adding or missing strokes), incorrect stroke sequencing, and poor grades in literacy courses. The directionality dimension consists of two items concerning the components of characters being upside down or reversed. The items for slow writing speed, inattention to handwriting tasks, messiness shown in homework or on test sheets, and failure to finish handwriting related tasks on time are included in the speed dimension. The items for the biomechanical characteristics of pencil grasp (e.g., pencil tip pressure on paper, tight grip) are included in the pencil grasp dimension. A higher score means poorer performance. The Taiwanese norm consists of handwriting scores of 468 typically developing children in grades 1 and 2, living in various geographic areas of Taiwan. Due to the absence of normality shown in the norm data, there is no transformation of standard scores provided, and only each percentage score compared to the norm is provided in the CHEF. A cut-off percentile of 15% was used for handwriting difficulty based on test manual recommendations [31]. The CHEF has acceptable to good internal consistency (Cronbach α = 0.70–0.93), test-retest reliability (r = 0.79–0.90), and split-half reliability (r = 0.64–0.98). The construct, discriminative, and concurrent validities of the CHEF have been validated [23, 31, 32]. Only the inter-rater reliability of the CHEF has not been investigated because there is only one class teacher in a class who teaches most courses, such as literacy and mathematics, in elementary schools in Taiwan.

#### Test of Nonverbal Intelligence, Third Edition (TONI-3)

The TONI-3 is a language-free intelligence test used to measure individual nonverbal abstract/figure problem solving ability [33]. The Chinese version of the TONI-3 with 62 item questions is for children aged from 7 years 6 months to 16 years 5 months. The child must choose a correct answer from 4–6 figures on each item question. The mean standard score of the norm is 100 with a standard deviation (SD) of 15. A standard score below 70 (-2SD) indicates intelligence disability. The Chinese version of the TONI-3 has been reported to be a valid and reliable test for school-aged students in Taiwan [34].

#### Movement Assessment Battery for Children—Second Edition (MABC-2)

There are 8 subtests to assess manual dexterity, ball skills, and balance in children 3–16 years of age in the MABC-2 [35]. For the purpose of this study, only the subtests for manual dexterity, including placing pegs, threading lace, and drawing a trail, were used. The mean standard score of the norm is 10, with a SD of 3. A lower standard score indicates poorer motor performance. A cut-off SD of 1 or 2 below the mean is usually used for screening children with a lack of motor coordination. Good validity and reliability have been demonstrated in the MABC-2 across cultures [36–38].

#### Test of visual perception skills—Third Edition (TVPS-3)

The TVPS-3 is a motor-free test that measures the visual perception of individuals aged between 4 and 18 years of age [39]. There are 7 subtests in the TVPS-3, including tests of visual discrimination, visual memory, spatial relationships, form constancy, sequential memory, figure-ground, and visual closure. Each subtest has 16 item questions. The child is asked to choose one out of 4–5 answer choices. The subtest is completed when the child incorrectly answers three consecutive items. The raw score for each subtest can be converted to scaled scores. The mean scaled score of the norm is 10 with an SD of 3. The sum of the scaled scores can be converted to a standard score that represents the overall performance. A mean of 100 and a SD of 15 represent a population’s distribution. A higher scaled or standard score indicates better visual-perceptual performance. Psychometric properties of the TVPS-3 have been investigated. For the test as a whole, reliability of internal consistency (coefficient α = 0.96) and test-retest reliability (r = 0.97) have been found satisfactory. Three types of validity inferences (content validity, criterion-related validity, construct validity) have been examined in the TVPS-3 [39].

#### Sensory Integration and Praxis Test (SIPT)—Finger identification (FI) and kinesthesia (KIN) subtests

The SIPT is a standardized measurement to assess various sensory processing functions in children at 4–8 years of age [40]. For the purpose of this study, the subtests of FI and KIN were used to evaluate the tactile and kinesthesia discrimination of the children under consideration. In the finger identification subtest, both of the child’s hands were placed on the table with the eyes occluded. The tester touched one finger in one or two different places or touched two fingers. Subsequently, the child opened his or her eyes and identified the touched fingers. A total of 16 trials were included in the subtest. In the kinesthesia subtest, the tester brought the child’s index finger from the first point on the testing paper to the second one and then returned to the first point with the child’s eyes occluded. Subsequently, the child moved his or her index finger to the second point from the first place by himself with closed eyes. The distance between the correct second point and the one the child moved to in the second place was recorded. The best 10 distances among 12 trials were averaged as the raw score. The standard score was provided through computerized scoring. The two subtests have been shown to have good discriminative validity. The test-retest reliability (FI = 0.74, KIN = 0.50) and inter-rater reliability (FI = 0.95, KIN = 0.99) are acceptable to good [40]. The tester had been trained by a qualified administer of the SIPT before data collection.

#### The Swanson, Nolan, and Pelham IV Scale (SNAP IV)

Based on the diagnostic criteria described in the Diagnostic and Statistical Manual of Mental Disorders IV (DSM-IV), the three subscales (26 questions) in the SNAP-IV were designed to evaluate the severity of inattention, hyperactive/impulsive, and appositive behaviors in children [41]. The Taiwanese norms for grades 1 to 8 for the Chinese version of SNAP IV have been established [42]. Mothers were asked to report the frequency of inattention, hyperactivity, and appositive behaviors of their children in previous weeks using a 4-point Likert scale from 0 (never) to 3 (very frequently) in the parental form of the SNAP IV- Chinese version. A higher score indicates greater severity of the behavior. Only the scores for the inattention subscale (9 questions) were analyzed in this study. The cut-off score for inattention for Taiwanese children in grade two is 12.5 (-1SD) and 17.1 (-2SD) for boys; 11.1 (-1SD) and 15.5 (-2SD) for girls. The internal consistency (Cronbach’s α = 0.88) and test-retest reliability (r = 0.73) of the inattention subscale are satisfactory. The inattention subscale has good concurrent validity with the inattention subscales of the Conners’ Parent Rating Scale (r = 0.79) and the Child Behavior Checklist (r = 0.70) [42].

### Procedure

The parents who agreed to participate were asked to complete a demographic questionnaire. The class teacher of the child was asked to complete the CHEF. As mentioned before, class teachers in elementary schools teach the majority of the courses. Hence, the class teacher is the person who most frequently and directly observes the child’s handwriting performance in a variety of handwriting tasks (far-point copying, near-point copying, dictation, and composition) in a natural environment. We asked the class teacher to observe the child’s handwriting performance when he/she filled out the CHEF. To avoid any bias, the parents of the preterm children were asked not to let the teacher know that their child was born prematurely.

Additionally, the preterm children and their parents were invited to come to our department for sensory, visual perceptual, fine-motor, and intelligence assessments. All children received the assessments in a quiet evaluation room of our department. The first author (Shih, HN), a certificated occupational therapist, performed all assessments. At the time of the evaluation, the children’s mothers were asked to fill out the SNAP IV-Chinese version.

### Statistical analysis

We classified the participants into four groups (preterm boys, preterm girls, full-term boys, and full-term girls) based on their gestational weeks at birth and sex. All analyses were performed using SPSS 17.0 software (SPSS Inc. Chicago, IL, USA). One-way analysis of variance (ANOVA) methods were used to compare the differences in demographic characteristics among the four groups. If the difference reached statistical significance, Bonferroni tests were used to further examine the differences between the two groups.

The effect of preterm birth (preterm vs. full term) and sex (boy vs. girl) on the CHEF scores was determined using a multivariate analysis of covariance (MANCOVA) with age at the time of the study and maternal socioeconomic status (SES) scores as covariates. Maternal SES scores were calculated using the levels of maternal education and occupation [43]. Independent t-tests were used to examine the difference between the two groups if the F values reached a significant difference. Fisher exact tests were used to analyze the group differences in the proportion of children with handwriting difficulty in each dimension of handwriting.

To identify predictors for handwriting performance in preterm children, Pearson correlation analyses were used to determine the association between each sensory, perceptual, and motor factor and handwriting dimensions. Subsequently, multiple linear regression analyses were used to determine the independent contribution of each significant factor to handwriting performance after adjusting for sex effects. For all analyses, the statistical significance was set as p < 0.05.

## Results

### Demographic characteristics

Parents of 139 children (68 preterm and 71 full-term children) agreed to participate in this study. Nine children were excluded because of failure to meet the criteria or invalid questionnaires. A total of 130 children (preterm: 63 and full-term: 67) ultimately were included in the analysis. Their demographic characteristics are shown in Table 1. There were no significant differences in demographic characteristics among the four groups of preterm boys, preterm girls, full-term boys, and full-term girls with the exception of gestational age (GA) and weight at birth, and maternal age. The majority of the preterm children (70%) were born moderate-to-late premature (32^+0^–36^+6^ weeks of gestation at birth). The average age of the mothers in the full-term girl group was significantly younger than those in the other three groups.

**Table 1:** The characteristics of preterm and full-term children in grade two.

	Preterm (n = 63)		Full-term (n = 67)		p
	Mean±SD or n (%)		Mean±SD or n (%)		
Child	Boys	Girls	Boys	Girls	
n	34	29	27	40	
Age (months)	96.3±3.2	95.7±2.4	96.5±3.8	95.2±3.8	0.333
GA at birth (weeks)	33.4±2.7	33.4±2.7	39.0±1.2	39.0±0.9	< 0.001
< 32^+0^	11 (32.4)	8 (27.6)	0	0	
32^+0^–33^+6^	5 (14.7)	7 (24.1)	0	0	
34^+0^–36^+6^	18 (52.9)	14 (48.3)	0	0	
Birth weight (gram)	2149±609	2050±655	3288±453	3116±313	< 0.001
Right handedness	30 (88.2)	28 (96.6)	26 (96.3)	38 (95.0)	0.459
Early intervention history	8 (23.5)	4 (13.8)	0	0	0.358
**Mother**					
Age (years)	39.2±3.4	40.5±5.3	38.7±3.6	36.3±4.3	0.001
Education (> 12 years)	25 (73.5)	18 (62.1)	17 (63.0)	22 (55.0)	0.435
Socioeconomic status score^a^	35.4±10.6	32.7±12.6	29.1±11.7	30.0±11.0	0.094

GA: gestational age.

^a^Socioeconomic status score = 4× educational level (level I-V) + 7× occupational level (level I-V). A higher score means higher socioeconomic status [43].

### Chinese handwriting performance in preterm and full-term children

The results of the CHEF are shown in Table 2. No main effect of group (preterm vs. full term) was found. A significant main effect of sex was found for the scores in the dimensions of construction (F = 20.03, p < 0.001), accuracy (F = 10.92, p = 0.002), and speed (F = 14.12, p < 0.001). The interaction effect for sex by group was significant in the speed dimension (F = 8.45, p = 0.003). Preterm boys had significantly higher scores (poorer performance) than full-term boys in the speed dimension (p = 0.008), whereas the two girl groups exhibited similar performance (p = 0.221). We further compared the performance of preterm and term boys in the four items of the speed dimension. After controlling for age and maternal SES, the MANCOVA results showed that preterm boys obtained higher scores (poorer) for the slower writing speed item than their peers (F = 8.461, p = 0.005) and were unable to maintain assignment sheet cleanly (F = 6.153, p = 0.016) and unable to complete handwriting-related assignments within a limited time (F = 7.174, p = 0.01).

**Table 2:** Handwriting performance of preterm and full-term children in grade two.

**CHEF score, mean**±**SD**	**Preterm**		**Full-term**		**p** **(group, sex, interaction)**
	**Boys** **(n = 34)**	**Girls** **(n = 29)**	**Boys** **(n = 27)**	**Girls** **(n = 40)**	
Construction	2.4±1.1	1.6±0.7	2.1±0.7	1.6±0.7	0.091, < 0.001, 0.219
Accuracy	2.4±1.2	1.7±0.7	2.0±0.8	1.9±0.9	0.294, 0.002, 0.079
Speed	2.7±1.1	1.6±0.8	1.9±0.8	1.9±0.9	0.074, < 0.001, 0.003
Pencil grasp	2.3±0.7	2.1±0.9	2.1±0.7	2.0±0.6	0.257, 0.117, 0.49
Directionality	1.7±0.9	1.4±0.6	1.5±0.7	1.6±0.8	0.839, 0.179, 0.11
	**Preterm**		**Full-term**		**p**
**CHEF score** ≤ **15% as handwriting difficulty, n (%)**	**Boys** **(n = 34)**	**Girls** **(n = 29)**	**Boys** **(n = 27)**	**Girls** **(n = 40)**	
Construction	11 (32.4)	2 (6.9)	2 (7.4)	2 (5.0)	0.004
Accuracy	8 (23.5)	2 (6.9)	1 (3.7)	4 (10.0)	0.104
Speed	7 (20.6)	2 (6.9)	2 (7.4)	4 (10.0)	0.337
Pencil grasp	3 (8.8)	3 (10.3)	2 (7.4)	1 (2.5)	0.565
Directionality	6 (17.6)	2 (6.9)	4 (14.8)	6 (15.0)	0.655

CHEF: Chinese Handwriting Evaluation Form.

The number of children with handwriting difficulty (≤ 15% of the norm) on each handwriting dimension of the CHEF is presented in Table 2. Overall, the preterm boy group had the greatest percent of children demonstrating difficulty in each handwriting dimension among the four groups with the exception of the pencil grasp dimension. However, only the difference in the construction dimension (p = 0.004) reached significance. A higher proportion of preterm boys (32.4%) exhibited difficulties in the construction dimension as compared to the other three groups (5.0–7.4%).

### Predictors of Chinese handwriting performance in preterm children

A subgroup of 39 preterm children (24 boys and 15 girls) and their parents agreed to come to our lab for sensory, perceptual, and motor evaluations. The demographic characteristics and handwriting performance of 39 preterm children were not shown to be significantly different from the other 24 preterm children whose parents refused to come for further assessments.

Of the 39 preterm children, only several children (n ≤ 2) had at-risk performance (1-2SD below the mean) in the TONI-3, SIPT-FI, and MABC-2 (manual dexterity subtests), but none of the children had dysfunctional performance (< 2SD below the mean) in these assessments (Table 3). However, a greater number of preterm children exhibited at-risk or dysfunctional performance in the TVPS-3 (12.8%), SIPT-KIN (15.4%) and SNAP IV (inattention subtest) (20.5%).

**Table 3:** Sensory, perceptual, and motor function in the preterm subgroup (n = 39).

Assessment	Mean±SD (range)	At risk^a^ n (%)	Dysfunction^b^ n (%)
TONI-3	101.2±11.3 (81–127)	1 (2.6)	0 (0)
TVPS-3	99.9±12.7 (76–131)	5 (12.8)	0 (0)
SIPT: Kinesthesia	-0.2±0.9 (-2.7–1.4)	3 (7.7)	3 (7.7)
SIPT: Finger identification	0.3±0.8 (-1.8–1.8)	2 (5.1)	0 (0)
MABC-2: Manual dexterity	15.0±3.5 (8–19)	0 (0)	0 (0)
SNAP IV: Inattention	8.4±4.5 (1–18)	7 (17.9)	1 (2.6)

TONI-3: Test of Nonverbal Intelligence—Third Edition, TVPS-3: Test of Visual Perception—Third Edition, SIPT: Sensory Integration and Praxis Test, MABC-2: Movement Assessment Battery for Children—Second Edition, SNAP IV: The Swanson, Nolan, and Pelham IV Scale.

^a^Scores are between 1 and 2SD below the mean of the norm.

^b^Scores are less than 2SD below the mean of the norm.

The results of the correlation analyses revealed that kinesthesia, manual dexterity, and inattention were significantly related to performance in various dimensions of handwriting performance (Table 4). The correlation between visual perception and performance in the construction dimension approached a significant difference (r = -0.302, p = 0.061). No factors were found to be related to the pencil grasp dimension. After controlling for sex, the results of the regression analyses revealed that inattention was the best predictor of a child’s performance in both the accuracy and speed dimensions (see Table 5). We did not find any factors predicting their performance in the construction and directionality dimensions.

**Table 4:** Correlates with handwriting performance in preterm subgroup (n = 39).

Variable	Construction	Accuracy	Speed	Pencil grasp	Directionality
**TONI-3**	-	-	-	-	-
**TVPS-3**	-	-	-	-	-
**SIPT: Kinesthesia**	-	-0.33*	-0.38*	-	-
**SIPT: Finger identification**	-	-	-	-	-
**MABC-2: Manual dexterity**	-0.34*	-0.33*	-	-	-
**SNAP IV: Inattention**	0.42**	0.46**	0.59***	-	0.32*

TONI-3: Test of Nonverbal Intelligence—Third Edition, TVPS-3: Test of Visual Perception—Third Edition, SIPT: Sensory Integration and Praxis Test, MABC-2: Movement Assessment Battery for Children—Second Edition, SNAP IV: The Swanson, Nolan, and Pelham IV Scale. “-” indicates the correlation did not reach statistical significance.

*p < 0.05.

**p < 0.01.

***p < 0.001.

**Table 5:** Predictors of handwriting performance in preterm subgroup (n = 39).

Predictor	Adjusted linear model^a^	
	Standardized β	p
**CHEF: Construction**		
MABC-2: Manual dexterity	-0.218	0.159
SNAP IV: Inattention	0.262	0.107
**CHEF: Accuracy**		
SIPT: Kinesthesia	-0.126	0.485
MABC-2: Manual dexterity	-0.155	0.35
SNAP IV: Inattention	0.334	0.046*
**CHEF: Speed**		
SIPT: Kinesthesia	-0.097	0.5
SNAP IV: Inattention	0.465	0.001**
**CHEF: Directionality**		
SNAP IV: Inattention	0.3	0.079

CHEF: Chinese Handwriting Evaluation Form, SIPT: Sensory Integration and Praxis Test, MABC-2: Movement Assessment Battery for Children—Second Edition, SNAP IV: The Swanson, Nolan, and Pelham IV Scale.

^a^Adjusted for sex

*p < 0.05.

**p < 0.01.

## Discussion

In this study, we investigated the handwriting performance of preterm- and full-term children in grade two using a multiple-dimension Chinese handwriting questionnaire. Thus, we could further classify handwriting legibility into construction, accuracy, and directionality. Our results for the second graders did not totally support the findings of Feder et al. in first graders [11] because we did not find the handwriting performance of the entire preterm group to be significantly worse than that of full-term children in grade two. One explanation may be the improvement that occurs in preterm children with handwriting problems from grade one to two. The findings demonstrated in the studies on this topic, in particular, show that children who are at-risk of or who are having difficulty with handwriting continue to improve in handwriting in the early grades [14, 15]. An alternative explanation is a different range of GA at birth in the preterm samples of these two studies. The preterm children included in Feder’s study were born at younger ages (< 34 weeks of gestation) than the children (< 37 weeks of gestation) in our study. The prematurity level may have thus led to this difference. Due to the heterogeneity of the preterm population, a longitudinal study with a large sample is required to specifically identify the influence of academic grades and prematurity levels on handwriting performance.

Consistent with the findings of male vulnerability to preterm birth in developmental outcomes and brain function, our results have also revealed a sex difference in handwriting performance. Preterm boys performed more poorly in the speed dimension of the CHEF than full-term boys, whereas preterm girls had comparable handwriting performance to that of full-term girls. In the dimensions related to legibility, similarly, a higher percentage of preterm boys had difficulty in the construction dimension compared to the other peer groups. In the accuracy dimension, although the group difference did not reach a statistical difference, a similar trend was found. As to the directionality dimension, there was no significant group and sex difference in the percentage of children with this difficulty. The current findings confirm the findings in Feder et al.’s study suggesting that preterm boys had worse legibility than preterm girls [11]. In addition, based on our findings, poor character construction may be the main cause for illegible handwriting in preterm boys, followed by accuracy problems. In contrast, the problems in the directionality dimension, such as reversal of components or mirror-writing, may be not a common factor contributing to their illegible handwriting.

In the current study, the correlation results revealed an association of kinesthesia, fine motor, and inattention with handwriting performance in preterm children. However, through the multiple regression analyses, inattention best predicted the performance in both the speed and accuracy dimensions in preterm children. In the past, attention has not often been measured and considered in handwriting studies. The current results suggest that the co-occurring deficits of attention and other handwriting components (e.g., fine motor, VMI) should be noted while identifying underlying factors for handwriting problems in clinical child populations. The results also imply that the enhancement of attention through direct intervention or environmental adaptation (e.g. decreasing environmental distractions during writing tasks) may be more helpful to improve the handwriting performance of the preterm population than only providing them with sensory-perceptual-motor intervention. Furthermore, evidence has indicated that preterm children are at high risk for attention problems [44, 45]. Thus, early screening and intervention for attention problems in preschoolers born prematurely may be essential to prevent handwriting problems later in elementary school.

We did not find an independent contribution of fine-motor skills to writing speed in the present preterm sample, which is contrast to the findings reported by Feder et al. (2005). Two explanations may be given for the lack of consistency. Firstly, all of the preterm children in the current study performed within the normal limit in the manual dexterity subtests (placing pegs, threading lace, tracing a path) of the MABC-2. Limited variations in fine-motor performance in our sample may have contributed to low power to detect a significant association with writing speed. Secondly, distinct aspects of fine-motor skills measured in both studies may have led to conflicting findings. In the study by Feder et al., (2005), a variety of fine motor assessments, including the fine motor subtests of the Bruininks-Osteretsky Test of Motor Proficiency, Motor Accuracy of the SIPT, Steadiness Test, and the subtests of Rotation (rotate a cube 180° in fingertips) and Translation (tasks of palm-to-fingers) of the In-hand Manipulation Skill Test, were administrated. Eventually, the regression results revealed that “Translation of the In-hand Manipulation Skill Test” was only important predictor of the handwriting speed tasks [11]. Therefore, the combined findings of both studies imply that the ability of in-hand manipulation may be more strongly related to handwriting speed than other aspects of fine motor skills in preterm children.

Feder et al. (2005) found a significant relation of visual perception and legibility in first graders born prematurely [11]. However, we did not find such a result in our preterm sample in grade two. One study indicated that the relationship of handwriting components to handwriting legibility varies in different age groups [27], which may help to explain the distinct findings of both studies. Similarly, a lack of association between visual perception and handwriting legibility has also been shown in typically developing children [20, 27] and those with developmental coordination disorders [29] in grade 2 or higher. The results from these studies suggest that the influence of visual perception on legibility may be important in new handwriting learners and less obvious with increasing age.

There were several limitations in this study. Firstly, due to recruitment difficulties (e.g., about 52.8% of teachers refused to fill out additional questionnaires on students other than the preterm participant in our pilot study); full-term controls were randomly selected from the second-grade classes of elementary schools in Tainan City rather than from the classmates of the preterm participants. Although there may be differences in learning curricula and handwriting practice across the teachers, the Curriculum Outlines for literacy learning in the elementary schools (e.g., the hours of courses for a week, learning goals for each academic grade) (i.e., the Model of K-12 Curriculum) [46] and the Handwriting Instructional Manual for teachers [47] published by the Ministry of Education in Taiwan should have made the differences minimal. Secondly, a few limitations involved the CHEF. Although the inter-rater reliability of the CHEF has not been investigated, other psychometric properties have been validated to support its reliability. It is suggested that the inter-rater reliability of the CHEF can be established by examining the correlation of the results of the CHEF rating by mothers familiar with their child’s handwriting performance and by teachers. On the other hand, due to the fact that the psychometric properties and norm of the CHEF was only validated in first and second graders, children in grade two were the participants in this study. The current findings may be generalized to children in higher grades with caution.

## Conclusions

The present results indicated sex-different performance in Chinese handwriting in preterm children without developmental disabilities. Preterm boys performed the worst in handwriting performance, particularly in the speed and construction dimensions, whereas the performance of preterm girls was comparable to that of full-term girls. Of the handwriting components, we found that attention is the best predictor of the performance in the speed and accuracy dimensions in preterm children. Therefore, an attention assessment is strongly suggested for preterm children with handwriting problems. Moreover, early screening and intervention for inattention at the preschool level may help enhance later handwriting performance in preterm children.

## Supporting information

S1 File (XLSX)
